# Simulated Gastrointestinal Digestion of Chestnut (*Castanea sativa* Mill.) Shell Extract Prepared by Subcritical Water Extraction: Bioaccessibility, Bioactivity, and Intestinal Permeability by In Vitro Assays

**DOI:** 10.3390/antiox12071414

**Published:** 2023-07-12

**Authors:** Diana Pinto, Ana Margarida Silva, Stefano Dall’Acqua, Stefania Sut, Anna Vallverdú-Queralt, Cristina Delerue-Matos, Francisca Rodrigues

**Affiliations:** 1 REQUIMTE/LAQV, ISEP, Polytechnic of Porto, Rua Dr. António Bernardino de Almeida, 4249-015 Porto, Portugal; diana.pinto@graq.isep.ipp.pt (D.P.); ana.silva@graq.isep.ipp.pt (A.M.S.); cmm@isep.ipp.pt (C.D.-M.); 2 Department of Pharmaceutical and Pharmacological Sciences, University of Padova, Via Marzolo 5, 35121 Padova, Italy; stefano.dallacqua@unipd.it (S.D.); stefania.sut@unipd.it (S.S.); 3 Nutrition, Food Science and Gastronomy Department, School of Pharmacy and Food Science, University of Barcelona, 08028 Barcelona, Spain; avallverdu@ub.edu; 4 Consorcio CIBER, M.P. Fisiopatología de la Obesidad y la Nutrición (CIBERObn), Instituto de Salud Carlos III (ISCIII), 28029 Madrid, Spain

**Keywords:** *Castanea sativa*, in vitro digestion, phenolic compounds, intestinal cell model, biological properties

## Abstract

Chestnut shells (CSs) are an appealing source of bioactive molecules, and constitute a popular research topic. This study explores the effects of in vitro gastrointestinal digestion and intestinal permeability on the bioaccessibility and bioactivity of polyphenols from CS extract prepared by subcritical water extraction (SWE). The results unveiled higher phenolic concentrations retained after gastric and intestinal digestion. The bioaccessibility and antioxidant/antiradical properties were enhanced in the following order: oral < gastric ≤ intestinal digests, attaining 40% of the maximum bioaccessibility. Ellagic acid was the main polyphenol in the digested and undigested extract, while pyrogallol–protocatechuic acid derivative was only quantified in the digests. The CS extract revealed potential mild hypoglycemic (<25%) and neuroprotective (<75%) properties before and after in vitro digestion, along with upmodulating the antioxidant enzymes’ activities and downregulating the lipid peroxidation. The intestinal permeation of ellagic acid achieved 22.89% after 240 min. This study highlighted the efficacy of the CS extract on the delivery of polyphenols, sustaining its promising use as nutraceutical ingredient.

## 1. Introduction

Chestnut (*Castanea sativa* Mill.) is a promising crop that is commercially exploited for food purposes due to its peculiar sensory properties and interesting nutritional value. The emerging demand for chestnut fruit has aroused the curiosity of food researchers in the search for new alternatives for the exploitation of chestnut by-products. Furthermore, the rise of the chestnut processing industry has also intensified the production of by-products, mainly shells that represent more than 66 tons just in Europe [1]. Chestnut shells (CSs) are of significant interest for different industries as prominent sources of bioactive molecules (particularly polyphenols and vitamin E) with exceptional pro-healthy benefits, such as the antioxidant, antimicrobial, and anti-inflammatory properties proven by in vitro and in vivo assays [1,2,3,4]. Notwithstanding, this plentiful biowaste remains unexplored, despite its richness in natural antioxidants that comprise a protective strategy to detoxify the human body of reactive oxygen and nitrogen species (ROS and RNS, respectively) and stimulate the antioxidant enzymes’ activity [3]. In previous studies, Pinto et al. [5,6,7] screened a new nutraceutical phenolics-rich ingredient extracted from CSs by an eco-friendly technology (i.e., subcritical water extraction (SWE)), proving its efficacy as an antioxidant as well as its safeness on intestinal cells. Ellagic acid, protocatechuic acid, pyrogallol, and gallic acid were the main phenolic compounds identified [7]. The valorization of CSs arising from the chestnut industrial production to the market as a bioactive ingredient for functional foods and nutraceuticals encompasses a valuable opportunity for agro-industries [8,9]. Nevertheless, the reuse of CSs as an antioxidant-rich ingredient also presents multiple challenges in industrial applications [1]. Even though the in vitro bioactivity and phytochemical composition of CSs have been extensively studied in recent years, a deep understanding of the biological fate of the phenolic compounds is vital to explore their potential bioactivity in vivo.

Healthy foods and related ingredients effectively ameliorate physical and mental disorders and protect against aging and chronic diseases (e.g., cardiovascular, metabolic, and neurological pathologies, as well as cancer), encouraging the design of greener formulations enriched with natural antioxidants, such as phenolic compounds. The bioactivity of phenolic compounds relies on the variety of their chemical structures and their interaction with other biomolecules (i.e., enzymes) or cell receptors (i.e., membrane transporters) [10]. Beyond their free form, phenolics may be covalently bound to indigestible constituents (e.g., dietary fiber) in the food matrix, which are metabolized into smaller molecules and further absorbed in the small intestine [11]. At this point, it is imperative to highlight that bioaccessibility (defined as the amount of nutrient released from the food matrix into the gastrointestinal tract) influences bioactivity and bioavailability (defined as the amount of available nutrient that can be effectively absorbed) [10,11]. Despite in vitro antioxidant assays being efficiently employed in non-digested foods for screening purposes, these outcomes may overestimate the biological role of complex matrices compared with in vivo studies that are expensive, time-consuming, and ethically questionable [10]. Hence, in vitro simulated digestion and intestinal permeability cell models have been applied to mimic human physiological conditions and predict the bioaccessibility and bioactivity of nutrients.

This study attempts, for the first time, to investigate the effects of in vitro gastrointestinal digestion and intestinal permeability in the bioaccessibility and bioactivity of phenolic compounds extracted from CSs by SWE, exploring its valorization as a novel nutraceutical ingredient. The total phenolic and flavonoid contents (TPC and TFC, respectively), antioxidant/antiradical activity, ROS and RNS scavenging potential, phenolic profile, and α-amylase and acetylcholinesterase (AChE) inhibition were assessed before and after in vitro digestion, while the phenolic composition was screened after the intestinal permeability assay. Additionally, multivariate data analysis was performed to outline the differences between digested and undigested samples.

## 2. Materials and Methods

### 2.1. Chemicals

All chemical reagents, solvents, and standards were of analytical reagent grade, used as received or dried by standard procedures, and acquired from commercial sources. Dulbecco’s modified Eagle medium (DMEM), fetal bovine serum (FBS), Hank’s balanced salt solution (HBSS), non-essential amino acids, penicillin, streptomycin, and trypsin–EDTA were supplied by Invitrogen Corporation (Life Technologies, S.A., Madrid, Spain). Triton X-100 was delivered by Sigma Chemical Co. (St. Louis, MO, USA), whereas dimethylsulfoxide (DMSO) was purchased from AppliChem (Darmstadt, Germany). 3-(4,5-Dimethyl-2-thiazolyl)-2,5-diphenyl-2H-tetrazolium bromide (MTT) reagent was purchased from Sigma-Aldrich (Steinheim, Germany). All other chemicals were obtained from Merck (Darmstadt, Germany).

### 2.2. Sample 

Chestnut shells were kindly provided by Sortegel (Sortes, Bragança, Portugal) in October 2018. Shells were dehydrated at 40 °C for 24 h (Excalibur Food Dehydrator, CA, USA) and ground to a particle size of 1 mm using an ultra-centrifugal grinder (Retsch ZM200, Düsseldorf, Germany). Finally, the powdered samples were stored in the dark at room temperature until extraction.

### 2.3. Preparation of C. sativa Shells Extract by Subcritical Water Extraction

SWE was conducted on CSs according to Pinto et al. [7]. The extraction was performed at 220 °C and 40 bar for 30 min, using a 400 mL Parr Reactor (Series 4560 high-pressure mini-reactors, Parr Instrument Company, Moline, IL, USA) attached to a Parr Reactor Controller (Series 4848, Parr Instrument Company, Moline, IL, USA). Briefly, powdered CSs (10 g) were mixed with deionized water (100 mL). A four-blade impeller at 200 RPM promoted the continuous agitation of the sample during extraction. Afterwards, the extract was filtered through Whatman n° 1 paper, centrifuged at 8000 rpm for 5 min (Sigma 3-30KS, Sigma, Osterode am Harz, Germany), and lyophilized (Telstar, model Cryodos-80, Barcelona, Spain). The final extract was stored at 4 °C until further analyses were performed. The extraction yield was 7.87 ± 0.37% (*w*/*w*) as previously reported by Pinto et al. [7].

### 2.4. In Vitro Simulated Gastrointestinal Digestion

In vitro simulated digestion was performed in subsequential phases following the procedure validated by Minekus et al. [12], with minor modifications [8]. For the oral digestion, lyophilized CS extract (50 mg/mL) was mixed with simulated salivary fluid at a 1:1 (*w*/*v*) ratio containing salivary α-amylase (75 U/mL) at pH 7 and incubated at 37 °C for 2 min in a water bath with stirring. After 2 min, aliquots of oral digest were collected. For the gastric digestion, the remaining oral digest was mixed with simulated gastric fluid at a 1:1 (*v*/*v*) ratio containing pepsin (2000 U/mL) at pH 3 and the mixture was incubated at 37 °C for 2 h in a water bath under stirring. After 2 h, aliquots of gastric digest were collected. For the intestinal digestion, the remaining gastric digest was merged with simulated intestinal fluid at a 1:1 (*v*/*v*) ratio containing pancreatin (100 U/mL) and bile (10 mM) at pH 7. The mixture was then incubated at 37 °C for 2 h in a water bath under stirring. After 2 h, aliquots of intestinal digest were collected, and the digestion process was ended. The aliquots collected at the end of each digestion phase were then centrifuged at 10,000× *g* for 10 min, and stored at −80 °C. KCl, KH_2_PO_4_, NaHCO_3_, NaCl, MgCl_2_(H_2_O)_6_, and (NH_4_)_2_CO_3_ were used to prepare the simulated fluids [12]. Three independent experiments were performed for each phase. The phenolics recovery index was calculated following Equation (1):

Recovery (%) = (PC_DS_/PC_FC_) × 100 (1)

where PC_DS_ is the phenolics content in the digested sample and PC_FC_ is the phenolics content in the undigested extract. The bioaccessibility (%) corresponds to the recovery rate after all digestion phases.

### 2.5. Total Phenolic and Flavonoid Contents

The TPC and TFC were determined by Folin–Ciocalteu and aluminum chloride assays, respectively, as described by Pinto et al. [8]. Results were presented as mg of gallic acid equivalents (GAEs) per g of dry weight (DW) (mg GAE/g DW) and mg of catechin equivalents (CE) per g of DW (mg CE/g DW), respectively, for TPC and TFC.

### 2.6. In Vitro Antioxidant/Antiradical Activities

Three spectrophotometric assays were employed to evaluate the antioxidant/antiradical properties, ABTS and DPPH radicals scavenging assays and ferric reducing antioxidant power (FRAP), following the procedures described by Pinto et al. [8]. Results were presented as mg of ascorbic acid equivalents (AAEs) per g of DW (mg AAE/g DW), mg of Trolox equivalents (TEs) per g of DW (mg TE/g DW), and mg of ferrous sulphate equivalents (FSEs) per g of DW (mg FSE/g DW), respectively, for the ABTS, DPPH, and FRAP assays.

### 2.7. Reactive Oxygen and Nitrogen Species Counteracting Potential

The counteracting ability of the digested and undigested CS extract was evaluated according to Pinto et al. [13] against ROS and RNS, namely the superoxide anion radical (O_2_^•−^), hydrogen peroxide (H_2_O_2_), hypochlorous acid (HOCl), peroxyl radical (ROO^•^), and peroxynitrite (ONOO^−^) in the presence and absence of sodium bicarbonate (25 mM) to mimic the physiological CO_2_ conditions, using a Synergy HT Microplate Reader (BioTek Instruments, VT, USA). Catechin and gallic acid were used as positive controls. Results are presented as inhibition, in % or IC_50_ (µg/mL), except for the ROO^•^ scavenging assay whose results were expressed as mg of TE per g DW (mg TE/g DW).

### 2.8. Antioxidant Enzymes Activities and Lipid Peroxidation

The antioxidant enzymes activities, namely catalase (CAT), glutathione peroxidase (GSH-Px), and superoxide dismutase (SOD), were monitored using commercial enzymatic kits (Sigma-Aldrich, Steinheim, Germany). The lipid peroxidation (LPO) was evaluated by a commercial kit from Merck (Darmstadt, Germany).

### 2.9. In Vitro Biological Activities

#### 2.9.1. Acetylcholinesterase Activity Inhibition

The inhibitory effects of undigested extract (125 µg/mL) and its digests on AChE activity were screened, using a commercial kit from Sigma-Aldrich (St. Louis, MO, USA), through the formation of a colorimetric product (412 nm) from the reaction between 5,5′-dithiobis(2-nitrobenzoic acid) and thiocholine. Results are expressed as the inhibition percentage (%).

#### 2.9.2. Amylase Activity Inhibition

The anti-amylase activity of undigested CS extract (125 µg/mL) and its digests was tested using a commercial kit from Sigma-Aldrich (St. Louis, MO, USA) according to the manufacturer’s protocol. The amylase activity is proportional to the amount of substrate (ethylidene-pNP-G7) cleaved by amylase, producing a colorimetric product with a maximum absorbance at 405 nm. Nitrophenol was employed as standard. Results are expressed as the inhibition percentage (%).

### 2.10. Phenolic Profile by LC/DAD-ESI-MS

The phenolic composition was investigated by LC/DAD-ESI-MS following the procedure described by Pinto et al. [13], using an Agilent 1260 liquid chromatography (LC) equipment attached to a diode array detector (DAD), a electrospray ion (ESI) source operating in negative ion mode and a Varian 500-MS mass spectrometer (MS). The column used was an Agilent Eclipse C18 3 × 100 mm (3.5 µm) and the elution was ternary. The mobile phases were water with 1% formic acid (A), acetonitrile (B), methanol (C), and water with 0.1% formic acid (D) applied in the following gradients: 0–12 min, 5% A, 5% B and 90% D; 12–18 min, 5% A, 15% B, 20% C and 60% D; 18–20 min, 5% A, 70% B, 20% C and 5% D; and then, 5% A, 5% B and 90% D. The flow rate and the injection volume were 400 µL/min and 10 µL, respectively. Turbo data-depending scanning (TDDS) was used to attain the fragmentation patterns of the eluted compounds. The ultraviolet (UV) spectrum was also confirmed for the compounds identified in comparison with the respective standards and literature. For quantification purposes, ellagic acid, protocatechuic acid, and pyrogallol were used as standards. The results are presented as µg of each phenolic compound per gram on DW.

### 2.11. In Vitro Intestinal Permeability

Caco-2 (ATCC Number: HTB-37; ethnicity, Caucasian; age, 72 years; sex, male; tissue, colon) and HT29-MTX (ATCC Number: HTB-38; ethnicity, Caucasian; age, 44 years; sex, female; tissue, colon) cells were acquired from the American Type Culture Collection (ATCC, Manassas, VA, USA). Caco-2 and HT29-MTX cells were cultivated separately in tissue culture flasks (Orange Scientific, Belgium) using a complete medium, containing DMEM supplemented with 10% (*v*/*v*) FBS, 1% (*v*/*v*) *L*-glutamine, 1% (*v*/*v*) non-essential amino acids, and 1% (*v*/*v*) antibiotic–antimitotic mixture (100 U/mL penicillin and 100 U/mL streptomycin). The cells were maintained at 37 °C in an incubator with 5% CO_2_ environment (CellCulture^®^ CO_2_ Incubator, ESCO GB Ltd., Barnsley, UK), supplied with fresh medium and washed with the HBSS every 48 h. Cells were harvested at 90–95% confluence using trypsin. Following the co-culture model, Caco-2 (passage 20–21) and HT29-MTX cells (passage 58–59) were seeded in the apical compartment (density of 1 × 10^5^ cells/cm^2^) of 12-well Transwell^®^ plates (3 µm pore diameter, polycarbonate, 1.12 cm^2^) applying a ratio of 90:10. The transepithelial electrical resistance (TEER) was measured during cultivation using an EVOM epithelial voltmeter equipped with electrodes (World Precision Instruments, Sarasota, FL, USA) to monitor the cells’ monolayer integrity. After 21 days of cultivation, changing the medium every two days, the culture medium was removed, and the cell monolayers were washed twice with HBSS at 37 °C. An initial apical concentration of 1000 µg/mL CS extract dissolved in HBSS was used, considering the cell viability results on Caco-2 and HT29-MTX reported in our previous study [7]. At fixed times (0, 15, 30, 45, 60, 90, 120, 180, and 240 min), aliquots of 200 µL were collected from the basolateral compartment and the same volume of HBSS was added. The procedure was performed at 37 °C. A negative control with HBSS was analyzed. TEER measurements were also conducted during the entire experiment. Finally, the samples were analyzed by LC/DAD-ESI-MS following the methodology implemented by Silva et al. [14]. The experiment was performed in triplicate. The results are presented as the permeability percentage of each phenolic compound across the intestinal model barrier, at different times, from the donor to the recipient compartment. The apparent permeability coefficient (P_app_) was calculated using Equation (2) as follows:

P_app_ (cm/s) = (dQ/dt) × A × C_0_ (2)

where dQ is the amount of permeated compound (μg), dt is the time of collection (s), A is the diffusion Transwell^®^ area (cm^2^), and C_0_ is the initial concentration of the compound (μg/mL).

### 2.12. Statistical Analysis

Results are expressed as the mean ± standard deviation. For all in vitro assays, a total of nine replicates (namely, three replicates for each one of the three independent assays performed) were analyzed for each independent experiment of the same digestion phase. Regarding statistical analysis, at least six replicates were used for each independent experiment of the same digestion phase. The statistical analysis was conducted by one-way ANOVA and Tukey’s HSD test using IBM SPSS Statistics 24.0 software (Chicago, IL, USA). Significant results were denoted for *p* < 0.05. Principal component analysis (PCA) was conducted using the GraphPad Prism v9 software (La Jolla, CA, USA) to identify the tendencies among samples and the most significant variables influencing the samples’ clustering.

## 3. Results and Discussion

### 3.1. Total Phenolic and Flavonoids Contents

The TPC and TFC of CS extract prepared by SWE were estimated before and after in vitro simulated digestion (Table 1).

According to Table 1, the changes in the TPC and TFC of the CS extract were denoted before and after in vitro digestion, underlining that the phenolic and flavonoid contents varied according to the digestion phase (oral, gastric, and intestinal). The TPC was enhanced in the following order: oral < gastric < intestinal digests < undigested extract, with an increase of 45% and 11% after gastric and intestinal digestion, respectively, suggesting that the gastric conditions (namely the presence of pepsin and acidic pH) enabled a higher phenolic recovery released from the extract matrix to the digestion medium when compared to the intestinal digestion. This explanation was also corroborated by the non-significant results (*p* > 0.05) for gastric and intestinal digests. Otherwise, all three digests revealed significant differences (*p* < 0.05) when compared to the undigested extract.

Beyond phenolic compounds, CS extract is also rich in flavonoids. The TFC decreased significantly (*p* < 0.05) after the in vitro digestion. Nonetheless, a significant increase of 31% (*p* < 0.05) was disclosed after intestinal digestion, while similar results (*p* > 0.05) were achieved for oral and gastric digests. In contrast to phenolic compounds, flavonoids are more easily recovered after the intestinal digestion, highlighting that the presence of pancreatin and bile, along with neutral pH, accelerated the release of flavonoids from the extract matrix, which agrees with previous studies [15].

The decreases in TPC and TFC from the undigested extract to intestinal digestion were already expected, as demonstrated in previous studies for other fruits and by-products, mainly due to the instability of phenolic compounds to pH changes and digestive enzymes that promote their degradation or biotransformation into other phenolics [16,17]. The latest advances have also reported decreases in the TPCs of fruit and by-product extracts, namely guava fruit, purple rice, and blueberries, after in vitro digestion, with a higher phenolics recovery after intestinal digestion [18,19,20]. The present outcomes were also higher than those reported for the inner and outer shells of *Castanea molissima* before (21.24 and 23.64 mg GAE/100 mL, respectively, for the chestnut’s outer and inner shells) and after gastric (83.58 and 47.14 mg GAE/100 mL, respectively, for the chestnut’s outer and inner shells) and intestinal digestion (56.69 and 32.47 mg GAE/100 mL, respectively, for the chestnut’s outer and inner shells) [17].

### 3.2. Bioaccessibility

The phenolics recovery index achieved the lowest value after the oral digestion and, subsequently, increased after gastric and intestinal digestion, corroborating the TPC results (Table 1). However, no significant differences (*p* > 0.05) were attained between gastric and intestinal digests, reinforcing the hypothesis that gastric enzymes and acidic pH have a major influence on the recovery of most of the phenolic compounds from the CS extract, while a smaller fraction was additionally recovered during intestinal digestion owing to gut enzymes and neutral pH. Considering flavonoids, the recovery rates significantly increased (*p* < 0.05) after intestinal digestion, proposing a distinct pattern from TPC where flavonoids are retained in a higher extent under intestinal conditions. Additionally, similar flavonoids recoveries (*p* > 0.05) were achieved after oral and gastric phases.

The amount of phenolic compounds and flavonoids that are released from a food matrix during gastrointestinal digestion, becoming available for intestinal absorption, is described as bioaccessibility [21]. Thus, the bioaccessibility of phenolic compounds and flavonoids was estimated as almost 40%, corresponding to their recovery after all phases of in vitro digestion and underlining a satisfactory release profile of the bioactive compounds from the CS extract. Hence, the phenolic compounds from CS extract become more bioaccessible during in vitro digestion owing to an increase in their concentrations from oral to intestinal phases, which may probably affect their bioavailability and bioactivity. Notably, other molecules (e.g., micronutrients, pigments, pesticides, drugs, etc.) present in CSs can be extracted along with the phenolic compounds and, furthermore, interfere with their digestibility, exerting negative effects [22,23,24]. This may also explain the 30% of maximum bioaccessibility and reduction in the phenolic content and bioactivity of CS extract after in vitro digestion.

Furthermore, these results are in line with previous studies on Meghalayan cherry pomace, Thair rice bran, and olive wastewater extracts [16,25,26]. Notwithstanding, a high fraction of phenolics and flavonoids (≈60%) present in CS extract has still not recovered during digestion, encouraging the implementation of encapsulation techniques as useful strategies to improve their bioaccessibility, as already reported by Radünz et al. [27].

### 3.3. Effects of In Vitro Digestion on Antioxidant/Antiradical Activity

Antioxidant compounds have long been documented as extraordinary allies against premature aging and oxidative stress-mediated pathologies (e.g., neurodegenerative, cardiovascular, and metabolic diseases, cancer, and inflammatory disorders) [1]. The antioxidant/antiradical properties of CS extract before and after simulated digestion were estimated by in vitro assays.

As shown in Table 1, the antioxidant/antiradical properties were enhanced in the following order: oral < gastric < intestinal digests < undigested extract, outlining a higher antioxidant response for the undigested extract and an increase in the antioxidant effects during in vitro digestion. Additionally, similar patterns were disclosed for the three antioxidant assays.

Considering the FRAP assay, the antioxidant activity increased by 57% and 60%, respectively, after gastric and intestinal digestion, when compared to the oral phase. Only a 3% increase was observed from gastric to intestinal phases, proposing that most of the antioxidant compounds are recovered under gastric conditions or the compounds retained have higher antioxidant properties, which was also previously reported for roasted coffee beans [28]. Additionally, no significant differences (*p* > 0.05) were attained between gastric and intestinal digests. Otherwise, the FRAP results after gastric and intestinal digestion were significantly different (*p* < 0.05) from the oral digest and the undigested extract. Compared with the undigested extract, the FRAP response was almost five times lower after gastric and intestinal digestion, and almost eight times lower after oral digestion.

The ABTS radicals’ scavenging capacity also increased 3.5 times and 4.1 times, respectively, after gastric and intestinal digestion, when compared to the oral phase. A 17% increase was achieved from gastric to intestinal phases, although no significant differences (*p* > 0.05) were observed. The antiradical activity was 7-fold, 2-fold, and 1.7-fold lower, respectively, after oral, gastric, and intestinal digestion, when compared to the undigested extract. An identical behavior was observed in the DPPH radicals scavenging assay, with the antiradical activity increasing by 47% and 75%, respectively, after gastric and intestinal digestion, when compared to the oral phase. A 28% increase in the DPPH response was accomplished from gastric to intestinal stages. However, these results were not significantly different (*p* > 0.05). Compared with the undigested extract, the antiradical potential of the oral, gastric, and intestinal digests was, respectively, 4.2, 2.8, and 2.4 times lower, revealing significant differences (*p* < 0.05) between the three digests and the undigested extract.

As expected, the antioxidant/antiradical effects of undigested CS extracts were significantly higher (*p* < 0.05) than the respective digests, which agrees with previous studies on the digestibility of antioxidants from food matrices [18,19,20,28]. Among the digests, higher results on the antioxidant/antiradical assays were achieved after gastric and intestinal digestion, suggesting that higher concentrations of antioxidant compounds were retained in these digests, or the bioactive compounds retained presented better antioxidant/antiradical properties.

Overall, the antioxidant/antiradical results are noteworthy and in close agreement with the TPC results, highlighting the contribution of phenolic compounds to the antioxidant/antiradical properties of undigested and digested CS extract. A possible explanation encompasses the increase in phenolic hydroxyl groups released from monomers or aglycones under gastric digestion that provide better antioxidant properties, as proven by the significant increase in antioxidant/antiradical effects after gastric phase [28]. Oppositely, in the intestinal environment, some bioactive compounds may be biotransformed into smaller molecules with different antioxidant properties, explaining a mild increase in the antioxidant response after intestinal digestion [20].

The obtained results were even better than those reported for Chinese chestnut shells before (≈50 mg AAE/100 mL for both the chestnut’s outer and inner shells in DPPH assay) and after gastric (≈100 and ≈75 mg AAE/100 mL, respectively, for the chestnut’s outer and inner shells) and intestinal (≈90 and ≈70 mg AAE/100 mL, respectively, for the chestnut’s outer and inner shells) digestion [17]. Previous studies have also revealed an increase in the TPC, antioxidant/antiradical properties, and bioaccessibility of phenolic extracts during in vitro digestion [18,19,20].

### 3.4. In Vitro Radicals Scavenging Efficiency

CSs are exceptional sources of natural antioxidants (particularly polyphenols and vitamin E) with potential application as nutraceuticals, providing protection against oxidative stress-induced damages on biomolecules [1,5,6]. The results of the scavenging efficiency assays of undigested and digested CS extracts against ROS and RNS endogenously produced in human body are presented in Table 2.

As shown in Table 2, the scavenging proficiency enhanced in the following order: oral < gastric < intestinal digests < undigested extract, achieving the highest counteracting efficiency against HOCl and ONOO^−^.

Considering the O_2_^●−^ scavenging potential, the best quenchers were gallic acid and the undigested extract, without significant differences (*p* > 0.05) between them. Among digests, the intestinal digest achieved the highest efficiency, followed by gastric and oral digests. Significant differences (*p* < 0.05) were attained between all digests.

Regarding the H_2_O_2_ quenching assay, intestinal and gastric digests more efficiently scavenged this species than the oral digest. Nevertheless, the undigested extract disclosed higher H_2_O_2_ counteracting potential. Gallic acid achieved a 5-fold higher IC_50_ value when compared to catechin. Significantly different results (*p* < 0.05) were observed for all samples.

The HOCl scavenging potential of the undigested extract and its intestinal digest were the highest, while gastric and oral digests inhibited 60% and 23% the HOCl generation, respectively. Gallic acid displayed the lowest capacity to scavenge HOCl, when compared to undigested CS extract, while catechin was the best scavenger. All digests and undigested extracts attained significantly different outcomes (*p* < 0.05).

The ROO^●^ scavenging activity screened by the ORAC assay explores the preventive effects on LPO [13]. Catechin was the best ROO^●^ quencher, followed by gallic acid. The undigested extract and the digests from oral, gastric, and intestinal phases attained mild ROO^●^ scavenging abilities, without significant differences between them (*p* > 0.05).

The ONOO^−^ counteracting assay was performed in the absence and presence of NaHCO_3_ to mimic the physiological bicarbonate levels (≈25 mM) [13]. Some polyphenols exhibit a more significant decrease in the ONOO^−^ quenching ability in the presence of NaHCO_3_ (namely caffeic, ferulic, gallic, and *p*-coumaric acids), in contrast with others (such as catechins) [29]. The quenching efficiency against ONOO^−^ increased in the following manner: oral < gastric < intestinal digests < undigested extract, achieving better results in the tested medium with NaHCO_3_ than in its absence. All digests and the undigested extract disclosed significantly different results (*p* < 0.05).

The promising outcomes of radical scavenging assays may be ascribed to the phenolic composition of the CS extract and respective digests, namely ellagic acid, pyrogallol, and protocatechuic acid, which scavenging potential was already demonstrated in recent studies [29,30,31,32]. Additionally, previous studies reported identical outcomes for CS extracts prepared by other eco-friendly techniques [3,4,7,33]. The decrease in the CS extract scavenging efficiency after in vitro digestion was also corroborated by the previous results on the TPC, antioxidant/antiradical assays, and phenolic profiling. Several studies have already proven the effective role of phenolic compounds in the scavenging of radicals endogenously produced in the human body (such as ROS and RNS) and, for this reason, phenolic compounds have been studied as promising bioactive molecules to be used in the prevention/co-therapy of lifestyle chronic diseases triggered by oxidative stress [34,35,36]. Hence, the decrease in phenolic concentrations (attested by spectrophotometric assays and phenolic profiling by LC-DAD-ESI/MS) upon digestion may prompt a decrease in the radical scavenging efficiency, suggesting that most of the phenolic compounds from the CS extract were degraded after digestion and/or metabolized into phenolic metabolites, with reduced bioactivity compared to the parent compounds that originate them [6,37]. In addition, previous studies on the in vitro digestion of foods and by-products obtained similar outcomes, highlighting lower antiradical properties after digestion [38,39,40].

### 3.5. Modulation of Antioxidant Enzymes’ Activities and Lipid Peroxidation

Besides antioxidant properties by radical scavenging and ferric reducing potential, another important antioxidant mechanism encompasses the modulatory effects of the CS extract on antioxidant enzymes’ activities and protection against LPO. Table 3 presents the results of the effects of different digests and undigested CS extracts on antioxidant enzymes activities, namely SOD, CAT and GSH-Px, and LPO.

The CS extract after intestinal digestion led to the highest SOD activity, followed by the gastric digest. The oral digest induced negligible effects on SOD activity, while the undigested extract had no effect on SOD activity. Significantly different results (*p* < 0.05) were determined for the three digestion phases.

After the gastric and intestinal digestion of CS extract, the CAT activity was 2.6 times higher when compared to the oral digest, revealing no significant differences (*p* > 0.05). In contrast to the SOD assay, the undigested extract disclosed a significantly (*p* < 0.05) higher capacity to upmodulate CAT activity.

A similar response was observed for the GSH-Px activity with the intestinal digest unveiling the highest modulatory capacity among digested samples. The gastric and intestinal digests of the CS extract improved the GSH-Px activity with results which were 2-fold and 4-fold higher, respectively, when compared to the oral digest. However, no significant differences (*p* > 0.05) were determined between the oral, gastric, and intestinal digests, while the result of the undigested extract was significantly different (*p* < 0.05).

The LPO is a useful indicator of oxidative injuries in living tissues, playing a key role in the pathogenesis of several chronic diseases. The CS extract after intestinal digestion efficiently protected against LPO, disclosing the lowest result, followed by gastric digest, undigested extract, and, to a lesser extent, oral digest. Only gastric digest and undigested CS extract showed similar results (*p* > 0.05).

The results highlight an upmodulating effect of different digests and undigested CS extracts on antioxidant enzymes’ activities and a downmodulating effect on LPO, therefore outlining their antioxidant properties. In general, among digested samples, the intestinal and gastric digests more efficiently improved SOD, CAT, and GSH-Px activities, and offered protection against LPO. Likewise, Pinto et al. [5] proved the in vivo antioxidant activity in the rats’ blood serum, liver, and kidney after the oral treatment with a CS extract (50 and 100 mg/kg body weight) through the upmodulation of SOD, CAT, and GSH-Px activities and the prevention of LPO. The current study proposes a slow release of the antioxidant compounds from the CS extract matrix during digestion, probably retaining a higher concentration of antioxidants after gastric and intestinal digestion, which provides better antioxidant properties to these digests.

### 3.6. Inhibition of Acetylcholinesterase and α-Amylase Activities

**Figure 1:**
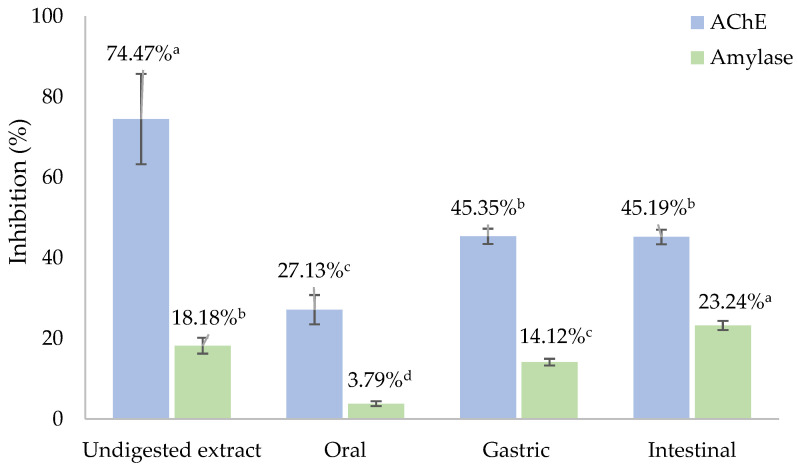
The inhibition of AChE and amylase activities of CS extract before and after in vitro digestion. Different letters denote significant differences (*p* < 0.05) between samples.

Beyond antioxidant effects, the phenolic compounds have demonstrated other remarkable biological activities, namely hypoglycemic and neuroprotective properties, with potential beneficial effects on metabolic and neurological diseases [4]. Figure 1 presents the results of the AChE and α-amylase inhibition of CS extract before and after digestion.

The undigested extract disclosed almost 75% of AChE inhibition at 125 µg/mL. This result was significantly different (*p* < 0.05) to its digested fractions. Despite the undigested extract attaining the highest result, the AChE inhibition improved during in vitro digestion. The CS extract after gastric and intestinal digestion effectively inhibited the AChE activity, without significant differences (*p* > 0.05). On the contrary, the oral digest attained the lowest inhibitory capacity. These outcomes are corroborated by the TPC results, highlighting the polyphenols’ role as entrapped in the extract matrix, which are slowly released during digestion (leading to the neuroprotective effects observed). It is noteworthy that similar results following the gastric and intestinal phases reinforced the fact that the phenolic compounds probably ascribed to the neuroprotective properties are mainly released under gastric conditions and preserved under intestinal conditions. Pinto et al. [4] reported lower results for the CS extract obtained by microwave-assisted extraction (MAE) with 26.42% inhibition at 250 µg/mL. In another study, Murugan et al. [41] reported a similar AChE inhibition for date palm (*Phoenix loureirii*) peduncle extracts after digestion (51.03–68.75%), which was attributed to its phenolic composition, namely catechin, chlorogenic acid, *p*-coumaric acid, ferulic acid, gallic acid, and rutin.

Considering the α-amylase activity, the undigested extract achieved 18% inhibition at 125 µg/mL. A significantly higher inhibitory effect (*p* < 0.05) was disclosed after intestinal digestion. The α-amylase inhibition potential raised during the in vitro digestion revealed mild hypoglycemic effects after gastric and intestinal digestion. Additionally, the inhibitory effect on α-amylase activity was negligible after the oral phase. Significant differences (*p* < 0.05) were attained between the undigested extract and its digests from oral, gastric, and intestinal phases. These results suggest that the polyphenols endowed with hypoglycemic properties are released from the extract matrix during digestion, achieving the maximum response under intestinal conditions, probably due to the polyphenols recovered in this phase and their hypoglycemic potential. Pinto et al. [4] reported a lower α-amylase inhibition for the CS extract prepared by MAE (7.81% at 125 µg/mL). Recently, Jagadeesan et al. [42] demonstrated an increase of up to 45% on the α-amylase and α-glucosidase inhibition for the green leafy vegetable *Allmania nodiflora* after the in vitro digestion, suggesting that caffeic acid, catechin, rutin, and saikosaponin were the main bioactive molecules responsible for this inhibitory effect.

The promising inhibitory effects observed may be ascribed to the phenolic compounds identified in the CS extract and its digests (particularly ellagic acid as well as pyrogallol and protocatechuic acid), which revealed mild AChE and α-amylase inhibitory potential, exerting potential neuroprotective and hypoglycemic effects [43,44,45]. The positive correlations of anti-AChE activity with TPC (*r*^2^ = 0.94) and TFC (*r*^2^ = 0.90), as well as anti-amylase activity with TPC (*r*^2^ = 0.43) and TFC (*r*^2^ = 0.35), emphasize the strong contribution of the phenolic compounds to the inhibitory activities observed for undigested and digested CS extract.

### 3.7. Phenolic Composition by LC-DAD-ESI/MS

**Figure 2:**
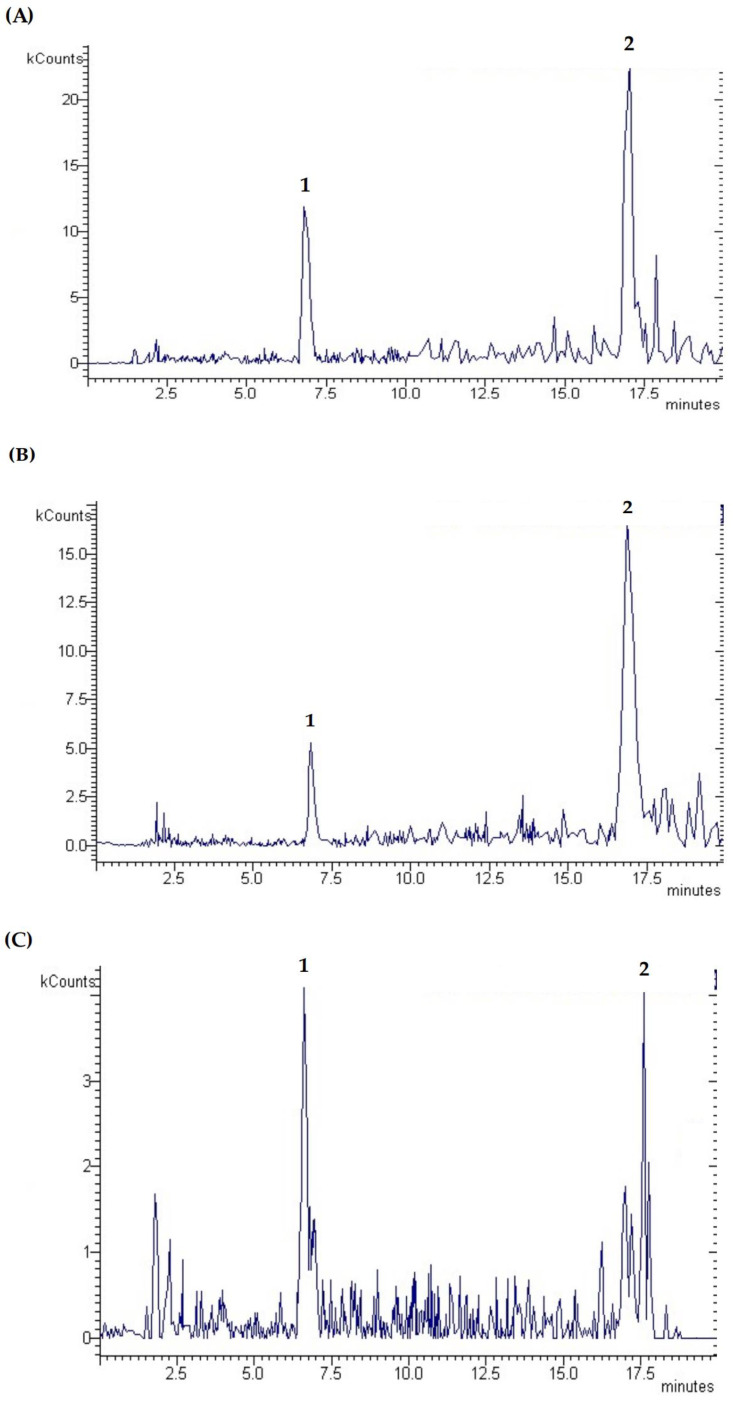
HPLC chromatograms of in vitro digestion samples from the CS extract prepared by SWE obtained after oral (**A**), gastric (**B**), and intestinal (**C**) phases. Peak identification is as follows: (1) ellagic acid; and (2) pyrogallol–protocatechuic acid derivative.

Phenolic compounds undergo biotransformation during metabolism, directly affecting their bioaccessibility, absorption, and bioactivity. Hence, in vitro and in vivo procedures have been designed to evaluate the metabolic pathways of phenolics upon digestion and the impact of the metabolites formed in the phenolics’ real bioactivity on human health [5]. Besides in vivo assays delivering a realistic outlook on the phenolics metabolism, these procedures are highly influenced by individual variation, drawbacks in samples pre-treatment and analytical methodologies, as well as possible interactions with other micro- or macronutrients [5,37]. In vitro simulated digestion models were implemented as simple and alternative approaches to appraise the phenolics metabolism, restraining the interindividual variability and interactions with food constituents. Considering the biological relevance, this study attempted to enlighten the real bioactivity of phenolic compounds from CSs upon human intake by comprehending the impact of digestive enzymes and pH changes from each digestion phase on the degradation and/or metabolization of phenolic compounds and, consequently, on their bioaccessibility using an in vitro digestion model. Figure 2 depicts the chromatograms of the CS extract after oral, gastric, and intestinal digestion. Table 4 summarizes the phenolic compounds in the CS extract before and after in vitro digestion.

The total content of phenolic compounds increased during digestion in the following order: intestinal (516.72 µg/g DW) = oral (640.00 µg/g DW) < gastric (907.32 µg/g DW) phases. These outcomes highlight changes in the phenolic composition of the CS extract during in vitro digestion, suggesting a significant impact of digestive enzymes and pH on the phenolic compounds retained in each phase by inducing molecular modifications. Only two phenolic compounds were identified and quantified in all digestion phases, namely ellagic acid and a pyrogallol–protocatechuic acid derivative. Ellagic acid was quantified in higher amounts in all digested fractions, compared to the pyrogallol–protocatechuic acid derivative that was not quantified in the undigested CS extract. Significant changes (*p* < 0.05) in the ellagic acid content were observed during in vitro digestion, with the highest amount determined under gastric conditions and the lowest in the intestinal digest. The undigested CS extract unveiled significantly higher amounts of ellagic acid, suggesting that this hydrolysable tannin underwent extensive metabolization through a digestive tract. Ellagic acid is one of the most abundant phenolic compounds reported in CSs [3,7,33,46]. Besides ellagic acid, some authors also identified ellagitannins (such as castalagin, vescalagin, and acutissimin), which may be hydrolyzed into ellagic acid at high temperatures or during digestion [4,47]. In addition, ellagic acid may be degraded and/or converted into metabolites during the digestion [48,49]. This may explain the decrease in ellagic acid content after intestinal digestion, suggesting its metabolization resulting from phase I and II reactions (e.g., dimethyl-ellagic acid), owing to the instability of ellagic acid under alkaline conditions and the action of digestive enzymes (namely pancreatin) and bile salts [5,50]. In addition, some phenolics could also bind to proteins in an alkaline environment via covalent, hydrophobic, and hydrogen bonds, reducing their concentrations [51,52].

Another peak, at a retention time of 5.63 min, presented significant UV absorption at 254 nm and a significant MS spectrum with confirming fragments, enabling a presumptive identification as a pyrogallol ether with protocatechuic acid. The MS fragments, presented in Table 4, indicate that this compound can cause water loss and the further loss of other fragments due to the retro-Diels–Alder reaction on aromatic rings [53]. The fragment at 137 allows the identification of the protocatechuic acid moiety esterified with a pyrogallol-type compound. In contrast to ellagic acid, the pyrogallol–protocatechuic acid derivative content was the lowest after gastric digestion, revealing no significant differences (*p* > 0.05) when compared to oral digest, while a significant increase of 87% (*p* < 0.05) was achieved from gastric to intestinal digestion. Indeed, gallic acid, previously identified in CS extracts, may be converted into pyrogallol via decarboxylation by thermal degradation (during extraction) or via human metabolism (during digestion), while protocatechuic acid is a catechin metabolite that results from the metabolism or thermal decomposition under extraction. These reactions may explain the presence of a pyrogallol–protocatechuic acid derivative in the digested samples, suggesting that these gallic acid and catechin metabolites may be conjugated into pyrogallol ether with protocatechuic acid. These phenolic metabolites have been proven to possess interesting pro-health effects [30,32,54]. Pyrogallol delivers antibacterial, antifungal, antioxidant, and antipsoriatic properties, while protocatechuic acid provides antioxidant, anti-inflammatory, anti-hyperglycemic, antimicrobial, and anti-apoptotic/pro-apoptotic activities [30,32]. For instance, Daré et al. [54] attested the capacity of protocatechuic acid and its alkyl esters, ethyl and heptyl protocatechuates, to counteract ultraviolet-induced oxidative damages in fibroblasts (L929 cells), preventing photoaging. These results highlight the formation of a pyrogallol–protocatechuic acid derivative during digestion since this compound was not identified in the undigested CS extract. In addition, its formation may result from the conjugation of pyrogallol and protocatechuic acid (previously identified in the CS extract or produced via metabolism of gallic acid and catechin) into an ether derivative compound. The increase in the pyrogallol–protocatechuic acid derivative during digestion indicates that intestinal conditions enable a higher recovery of this phenolic metabolite owing to the action of digestive enzymes (i.e., pancreatin), bile salts, the minerals composition of simulated biological fluid, and neutral pH [15]. Oppositely, the acidic pH and gastric enzymes hinder the hydrolysis of certain phenolic compounds (e.g., catechin and derivatives) into metabolites (e.g., protocatechuic acid), explaining the reduced concentration of the pyrogallol–protocatechuic acid derivative in the gastric phase [15].

The discrepancies between the phenolic profile and the in vitro bioactivity of the CS extract after intestinal digestion (which revealed the best outcomes in some assays, while disclosed lower results than the gastric digest in others) may be explained by the formation of metabolites from phenolic compounds during the intestinal digestion that were not identified, but strongly contribute to the potent antioxidant, neuroprotective, and hypoglycemic responses observed in most in vitro bioactivity assays [49,51]. Hence, metabolomic studies should be conducted to comprehend the structural modifications of phenolic compounds during digestion, and the metabolic pathways followed.

### 3.8. In Vitro Intestinal Permeability

**Figure 3:**
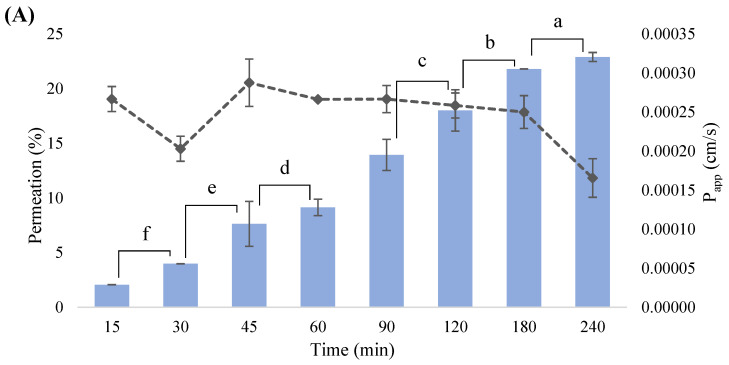

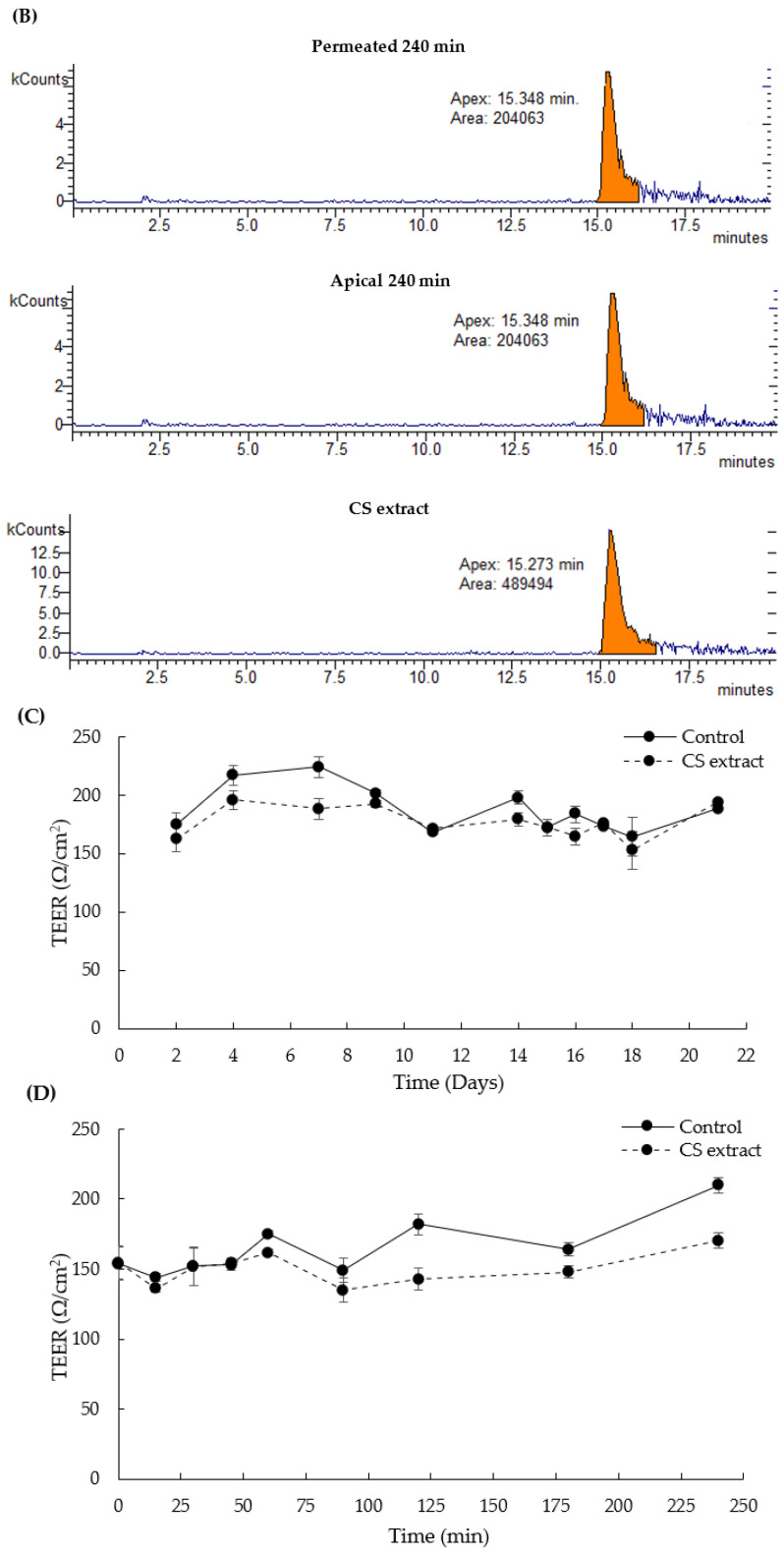
Permeation of ellagic acid (*m*/*z* 301; RT: 15.3 min) from CS extract across intestinal barrier at different time-points (**A**), peak chromatograms of permeated samples after 240 min, apical 240 min, and CS extract at 1 mg/mL (**B**), TEER values during 21 days of the co-culture model (**C**), and TEER values during the intestinal permeability assay (**D**). The same letter denotes non-significant differences (*p* > 0.05) between time points.

After metabolism, polyphenols and their metabolites undergo intestinal absorption. Considering the dynamism and complexity of the intestinal absorption process, several in vitro cell models (such as Caco-2/HT29-MTX co-culture) have been developed as promising strategies to simulate the human intestinal epithelium and accurately assess the phenolic permeability across the intestinal barrier [55]. The CS extract concentration of 1 mg/mL was selected for the intestinal permeability assay based on data previously published by our research group [5,7]. Our findings attested the in vitro bioactivity and safety of the CS extract at 1 mg/mL on the two intestinal cell lines used in the intestinal co-culture model (Caco-2 and HT29-MTX cells), sustaining the selection of this concentration to pursue the studies. Figure 3 presents the permeation of ellagic acid from the CS extract at different time-points.

Ellagic acid was the only phenolic compound identified and quantified in the permeated samples. The permeation rates steadily increased from 15 to 240 min, achieving a maximum intestinal permeability of 22.89% at the last time-point (240 min). P_app_ coefficient is a reliable indicator of the speed at which bioactive compounds pass through the intestinal membrane, predicting the intestinal transport mechanisms [55]. As shown in Figure 3A, the P_app_ results (2.66 × 10^−4^ and 1.65 × 10^−4^ cm/s, respectively, after 15 and 240 min of permeation) were similar to those reported by Akter et al. [56] for ellagic acid from the Kakadum plum (796 × 10^−6^ and 201 × 10^−6^ cm/s, respectively, after 120 and 30 min of permeation) using an identical in vitro model. The P_app_ values obtained were higher than 1 × 10^−6^ cm/s, indicating a substantially high permeation of ellagic acid across the intestinal barrier, enabling it to reach the target site and achieve the desirable therapeutic effects, particularly the oxidative protection. However, the low solubility of ellagic acid in aqueous solvents or the possible degradation and/or bioconversion into metabolites, such as (di)methyl-ellagic acid, may influence its intestinal absorption [5]. Given the promising broad therapeutic activities of ellagic acid, a deep understanding of its intestinal absorption is extremely important to appraise its potential in vivo bioactivity. The present outcomes corroborate the results of recent studies that detected ellagic acid and its methylated conjugate on in vitro and in vivo studies using intestinal cell models and animals [5,57,58,59]. For instance, Pinto et al. [5,6] conducted in vivo bioavailability studies that employed Wistar rats orally treated with CS extract (50 and 100 mg/kg b.w.), detecting ellagic acid and its methylated metabolite in blood serum and liver. In another study, Mao et al. [58] demonstrated the uptake of ellagic acid along with two other phenolic compounds, namely gallic acid and corilagin, from the *Fructus phyllanthi* extract tested at a concentration of 1 mg/mL across Caco-2 cell monolayers up to 180 min, reporting higher transport percentages for ellagic acid. Furthermore, Iglesias et al. [57] and Whitley et al. [59] attested to the permeation of ellagic acid across a Caco-2 cells monolayer, exploring their use to mitigate inflammatory bowel disease and prevent cancer in animals. These studies suggest that ellagic acid is effectively capable of permeating in vitro intestinal models, which was also determined in the present study [5,6,57,58,59]. Notwithstanding, any comparisons between the in vivo and in vitro results achieved in this study should be carefully established considering the limitations of both assays, particularly the difficulty of the in vitro assays in accurately mimicking the biological conditions.

Considering previous studies on the phytochemical composition of CSs, more phenolic compounds with poor intestinal permeability were probably retained in the intestinal cell barrier, hindering their passage to the basolateral compartment due to their complex chemical structure with multiple rings or biotransformation into metabolites that are difficult to identify due to the lack of standards and the presence of cellular components (e.g., amino acids), interfering with the detection methods [55,60]. Nevertheless, the phenolics retained within the cell membrane may also provide potential health benefits through cell cytoprotection, reducing the oxidative stress that is a mechanism involved in the transport of phenolic compounds and activated during the cell permeation [60]. It is also noteworthy to highlight that the initial CS extract concentration tested was 1 mg/mL (containing 10.50 µg/mg of ellagic acid), which may have limited the permeation of phenolic compounds through the intestinal barrier, preventing it from reaching values that are necessary to resemble the physiological effects due to the low concentration of phenolic compounds retained in the CS extract. However, the upgrading of the CS extract concentration was not possible considering the cell viability results (published in our previous study [7]) that ensure its safety only up to 1 mg/mL. The TEER was measured for 21 days of co-culture and during the experiment to guarantee the integrity of the cell barrier. According to Figure 3C, the TEER values regarding the CS extract increased until the 9th day (192.22 Ω/cm^2^), corroborating the cell growth. On the 11th day, the values slightly decreased (171.33 Ω/cm^2^), and then remained stable until the 18th day (152.56 Ω/cm^2^) and reached 194.14 Ω/cm^2^ on the 21st day. During the 240 min assay (Figure 3D), the TEER values maintained constant, ranging between 135.00 (at 90 min) and 170.67 Ω/cm^2^ (at 240 min). Similar values were reported by Silva et al. [14] to evaluate the permeation of kiwiberry leaf extract using an identical co-culture model. Nonetheless, these values were lower when compared to Caco-2 monolayers and triple co-culture (Caco-2/HT29-MTX/Raji B) models [55]. These discrepancies may be explained by the presence of mucus-secreting HT29-MTX cells, modulating the Caco-2 tight junctions and increasing the intercellular spaces between Caco-2 and HT29-MTX cells, which may influence the transport mechanisms of phenolic compounds across the intestinal barrier [55]. Nonetheless, this is the first study to assess the in vitro intestinal permeability of phenolic compounds from CSs using the Caco-2/HT29-MTX co-culture model.

Notably, the metabolism of nutrients in the gastrointestinal tract also relies on the intestinal microbiota composition and activity [61]. However, it was not possible to simulate the intestinal bacteria environment in the in vitro digestion and permeability assays, which was a drawback of these studies. Future studies should evaluate the impact of gut microbiota in the digestibility and metabolic fate of phenolics-rich CS extract.

### 3.9. Screening of Potential Differences by Multivariate Data Analysis

**Figure 4:**
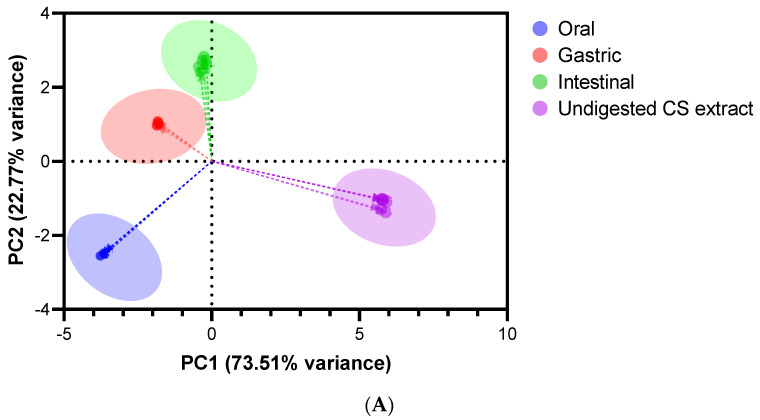

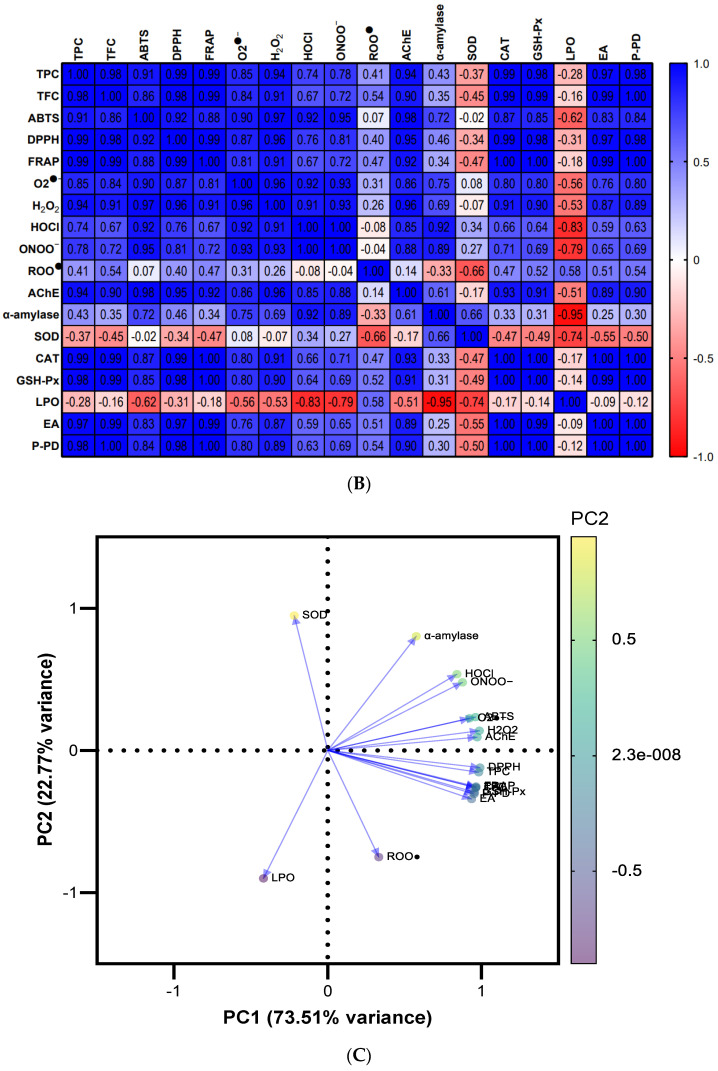
Multivariate data analysis on the in vitro bioactivity and phenolic composition of the digested and undigested CS extract: (**A**) scores plot by the digestion phase and undigested CS extract; (**B**) heatmap correlation diagram; and (**C**) the loadings plot of the variables under study.

PCA and heatmap correlation diagram were outlined to investigate the different trends between the undigested CS extract and its digested fractions (Figure 4).

The scores plot (Figure 4A) unveils four separated groups with 96.28% of the explained cumulative variance, highlighting evident differences on the bioactivity, bioaccessibility, and phenolic composition of digested fractions from oral, gastric, and intestinal phases, and undigested CS extract. The principal component (PC) 1 explains 73.51% of the results’ variance (eigenvalue = 21.35), while the remaining 22.77% of variance is explained by PC2 (eigenvalue = 6.30). The differences between oral digest and undigested extract are clearly explained by PC1, while the PC2 elucidates the variances between gastric and intestinal digests. In summary, gastric, and intestinal digests were more identical to each other, while the oral digest and undigested CS extract showed clearly distinct responses.

The heatmap correlation (Figure 4B) emphasizes the positive correlations of the TPC and TFC with ABTS, DPPH, FRAP, O_2_^●−^, H_2_O_2_, HOCl, ONOO^−^, AChE, CAT, and GSH-Px based on *r*^2^ above 0.67, highlighting that phenolic compounds seem to greatly contribute to the antioxidant/antiradical properties, ROS and RNS scavenging efficiency, upmodulating of antioxidant enzymes activities, and neuroprotective effects of the undigested CS extract and its oral, gastric, and intestinal digests. Additionally, weak positive correlations of α-amylase inhibition with TPC (*r*^2^ = 0.43) and TFC (*r*^2^ = 0.35) were also observed, pointing out the role of the phenolic compounds to the inhibitory capacity of the CS extract and its digests on α-amylase activity. Otherwise, negative correlations between the LPO and most studied variables underline that higher antioxidant/antiradical properties, radical scavenging capacity, and the upmodulating of CAT and GSH-Px activities were also associated with a reduction in LPO, offering protective effects against lipid peroxidation. Furthermore, ellagic acid and protocatechuic-pyrogallol derivative are proposed as the main phenolic compounds, contributing to the in vitro bioactivity of the digested and undigested CS extract (*r*^2^ > 0.51 and *r*^2^ > 0.54, respectively), apart from weak correlations with α-amylase (*r*^2^ < 0.30), negative correlations with SOD activity (*r*^2^ < −0.55), and no correlations with LPO.

The loadings plot (Figure 4C) corroborates the conclusions of the heatmap correlations, demonstrating that: (i) TPC, TFC, ABTS, DPPH, FRAP, O_2_^●−^, H_2_O_2_, HOCl, ONOO^−^, AChE, CAT, and GSH-Px responses are more positively correlated to each other; (ii) the ellagic acid and pyrogallol–protocatechuic acid derivatives highly contribute to all of these responses; (iii) ROO^●^, SOD, LPO, and α-amylase inhibition have distinct responses and are poorly correlated to each other and with the previously indicated variables.

The multivariate analyses highlighted a marked heterogeneity between the digested and undigested CS extract endorsed in the in vitro bioactivity and bioaccessibility of phenolic compounds. It is noteworthy that the phenolics’ concentration and their bioactivity may fluctuate in response to metabolic pathways that influence their bioaccessibility.

## 4. Conclusions

The current study provides novel insights into the effects of in vitro gastrointestinal digestion and intestinal permeability on the bioaccessibility and bioactivity of a phenolics-rich CS extract towards its valorization as an active ingredient for nutraceuticals and functional foods. The results demonstrated that the phenolic composition of the CS extract is affected by pH changes and the action of digestive enzymes during in vitro digestion, inducing the possible degradation of complex polyphenols and the modifications of their chemical structures and solubility. The antioxidant/antiradical properties, radical scavenging proficiency, and inhibitory effects on AChE and α-amylase activities improved during digestion in the following order: oral < gastric ≤ intestinal digests. The phenolic bioaccessibility increased during digestion (by up to 40%), suggesting a slow release of polyphenols entrapped into the extract matrix. Hence, the better outcomes in terms of gastric and intestinal digests are probably ascribed to the higher phenolic concentration retained after gastric and intestinal digestion. Considering the phenolic profile, ellagic acid was more efficiently recovered after gastric digestion, while its concentration significantly decreased after intestinal digestion. Only one metabolite, namely the pyrogallol–protocatechuic acid derivative, was detected after digestion, reaching higher concentrations in the intestinal digest. In addition, ellagic acid was the only polyphenol that permeated the in vitro intestinal cell model, reaching 23% of permeation. The promising contribution of polyphenols to the bioactivity of the CS extract upon digestion and intestinal permeability was reinforced by multivariate analysis, emphasizing clear differences between the digestion phases. Further studies should focus on metabolomic studies and the formulation of a nutraceutical product incorporating the CS extract. It is worth noting that, prior to the valorization of CSs for nutraceutical purposes, a careful assessment of the presence of harmful molecules (e.g., pesticides, drugs, metals, etc.) is of the utmost importance, considering that these compounds may negatively affect human health.

## Figures and Tables

**Table 1:** TPC, TFC, recovery rates of phenolics and flavonoids, antioxidant activity, antioxidant enzymes activities, and lipid peroxidation of CS extract prepared by SWE before and after in vitro digestion.

	In Vitro Simulated Digestion			Undigested CS Extract
	Oral Digest	Gastric Digest	Intestinal Digest	
**TPC (mg GAE/g DW)**	87.86 ± 3.06 ^c^	127.44 ± 5.34 ^b^	141.77 ± 12.81 ^b^	362.28 ± 21.10 ^a^
**Phenolics recovery (%)**	24.28 ± 1.02 ^b^	35.21 ± 1.29 ^a^	39.33 ± 5.16 ^a^	−
**TFC (mg CE/g DW)**	31.42 ± 1.02 ^c^	29.87 ± 1.06 ^c^	39.16 ± 2.08 ^b^	103.87 ± 3.74 ^a^
**Flavonoids recovery (%)**	30.26 ± 1.02 ^b^	28.78 ± 1.41 ^b^	37.72 ± 2.05 ^a^	−
**ABTS (mg AAE/g DW)**	104.36 ± 2.40 ^c^	366.45 ± 11.84 ^b^	427.64 ± 6.27 ^b^	728.52 ± 45.35 ^a^
**DPPH (mg TE/g DW)**	187.56 ± 4.16 ^c^	276.15 ± 28.29 ^b^	328.73 ± 10.10 ^b^	784.58 ± 29.02 ^a^
**FRAP (µmol FSE/g DW)**	979.02 ± 104.46 ^c^	1537.68 ± 87.14 ^b^	1562.75 ± 88.74 ^b^	7659.96 ± 94.23 ^a^

AAE, ascorbic acid equivalent. CE, catechin equivalent. DW, dry weight. FSE, ferrous sulfate equivalent. FRAP, ferric reducing antioxidant power. GAE, gallic acid equivalent. TE, Trolox equivalent. TFC, total flavonoid content. TPC, total phenolic content. Different letters (a–c) denote significant differences among samples (*p* < 0.05).

**Table 2:** The ROS and RNS scavenging capacity of undigested and digested CS extract prepared by SWE before and after in vitro digestion. IC_50_ is defined as the in vitro concentration needed to scavenge 50% of the pro-oxidant species in a tested medium (mean ± standard error of the mean). Different letters (a–c) denote significant differences (*p* < 0.05) between the IC_50_ results of positive controls and undigested extract. Different numbers (1–3) denote significant differences (*p* < 0.05) between the inhibition percentages of digests. * indicates results directly expressed as inhibition percentages tested in the samples. # indicates data published in our previous paper [7] (only stated in this table for comparison with digested fractions).

	Reactive Oxygen Species				Reactive Nitrogen Species	
	O_2_^●−^	H_2_O_2_	HOCl	ROO^●^	ONOO^−^	
	IC_50_ (µg/mL)			µmol TE/mg DW	In Presence of NaHCO_3_ IC_50_ (µg/mL)	In Absence of NaHCO_3_ IC_50_ (µg/mL)
**Oral digest**	9.88 ± 1.27 *^,3^	14.34 ± 2.28 *^,1^	22.70 ± 1.12 *^,3^	0.04 ± 0.01 ^b^	18.37 ± 0.42 *^,3^	16.40 ± 0.59 *^,3^
**Gastric digest**	17.85 ± 1.32 *^,2^	34.82 ± 3.11 *^,2^	60.17 ± 0.23 *^,2^	0.13 ± 0.00 ^b^	55.61 ± 1.21 *^,2^	50.81 ± 0.96 *^,2^
**Intestinal digest**	60.65 ± 2.07 *^,1^	56.59 ± 1.66 *^,3^	90.10 ± 0.85 *^,1^	0.21 ± 0.01 ^b^	83.72 ± 1.63 *^,1^	74.55 ± 0.54 *^,1^
**Undigested CS extract ^#^**	12.92 ± 0.34 ^#,b^	114.51 ± 3.63 ^a^	0.79 ± 0.06 ^#,b^	0.32 ± 0.01 ^#,b^	1.75 ± 0.07 ^a^	1.88 ± 0.04 ^a^
***Positive controls***						
**Catechin**	48.21 ± 4.79 ^a^	20.78 ± 0.75 ^c^	0.37 ± 0.01 ^c^	1.81 ± 0.12 ^a^	0.23 ± 0.01 ^b^	0.16 ± 0.02 ^b^
**Gallic acid**	10.95 ± 1.40 ^b^	106.03 ± 0.93 ^b^	1.81 ± 0.02 ^a^	1.08 ± 0.10 ^a^	0.29 ± 0.02 ^b^	0.15 ± 0.02 ^b^

**Table 3:** The effects of the undigested CS extract and its digests on antioxidant enzymes’ activities and lipid peroxidation.

	In Vitro Simulated Digestion			Undigested CS Extract
	Oral Digest	Gastric Digest	Intestinal Digest	
**CAT (nmol/min/g DW)**	80.64 ± 10.34 ^c^	213.42 ± 29.67 ^b^	208.13 ± 23.38 ^b^	1706.14 ± 44.96 ^a^
**GSH-Px (µmol/min/g DW)**	160.77 ± 5.68 ^b^	352.89 ± 21.60 ^b^	633.44 ± 13.64 ^b^	9083.60 ± 824.20 ^a^
**SOD (µmol/min/g DW)**	13.65 ± 1.89 ^c^	249.52 ± 10.71 ^b^	521.31 ± 42.83 ^a^	n.d.
**LPO (nmol MDA/mg DW)**	3.95 ± 0.27 ^a^	1.30 ± 0.09 ^b^	0.40 ± 0.02 ^c^	1.58 ± 0.06 ^b^

CAT, catalase. DW, dry weight. GSH-Px, glutathione peroxidase. MDA, malondialdehyde. LPO, lipid peroxidation. n.d., not determined. SOD, superoxide dismutase. Different letters (a–c) denote significant differences among samples (*p* < 0.05).

**Table 4:** Quantification of ellagic acid and pyrogallol–protocatechuic acid derivative in CS extract prepared by SWE before and after in vitro digestion. Different letters denote significant differences (*p* < 0.05) between in vitro digestion phases for the same compound. n.d.—not determined.

N°	Rt (min)	Compounds	[M−H]^−^	Fragments	Amount (µg/g DW)			Amount (µg/mg DW)
					Oral	Gastric	Intestinal	Undigested CS Extract
1	5.63	Pyrogallol–protocatechuic acid derivative	261.1	261 243 203 177 137	199.20 ± 16.97 ^b^	165.81 ± 22.35 ^b^	310.32 ± 28.68 ^a^	n.d.
2	16.8	Ellagic acid	301.0	301 284 257 229 185 157 129	440.80 ± 14.71 ^b^	741.50 ± 92.55 ^a^	206.40 ± 65.89 ^c^	10.50 ± 0.26

## Data Availability

The data used to support the findings of this study can be made available by the corresponding author upon request.
